# Supplementary material for: Quasi-essentiality of RNase Y in Bacillus subtilis is caused by its critical role in the control of mRNA homeostasis
Source: Nucleic Acids Res. 2021 Jun 22;49(12):7088–102. doi: 10.1093/nar/gkab528 (PMC8266666; doi:10.1093/nar/gkab528)
Supplement: gkab528_Supplemental_File [file gkab528_supplemental_file.pdf]

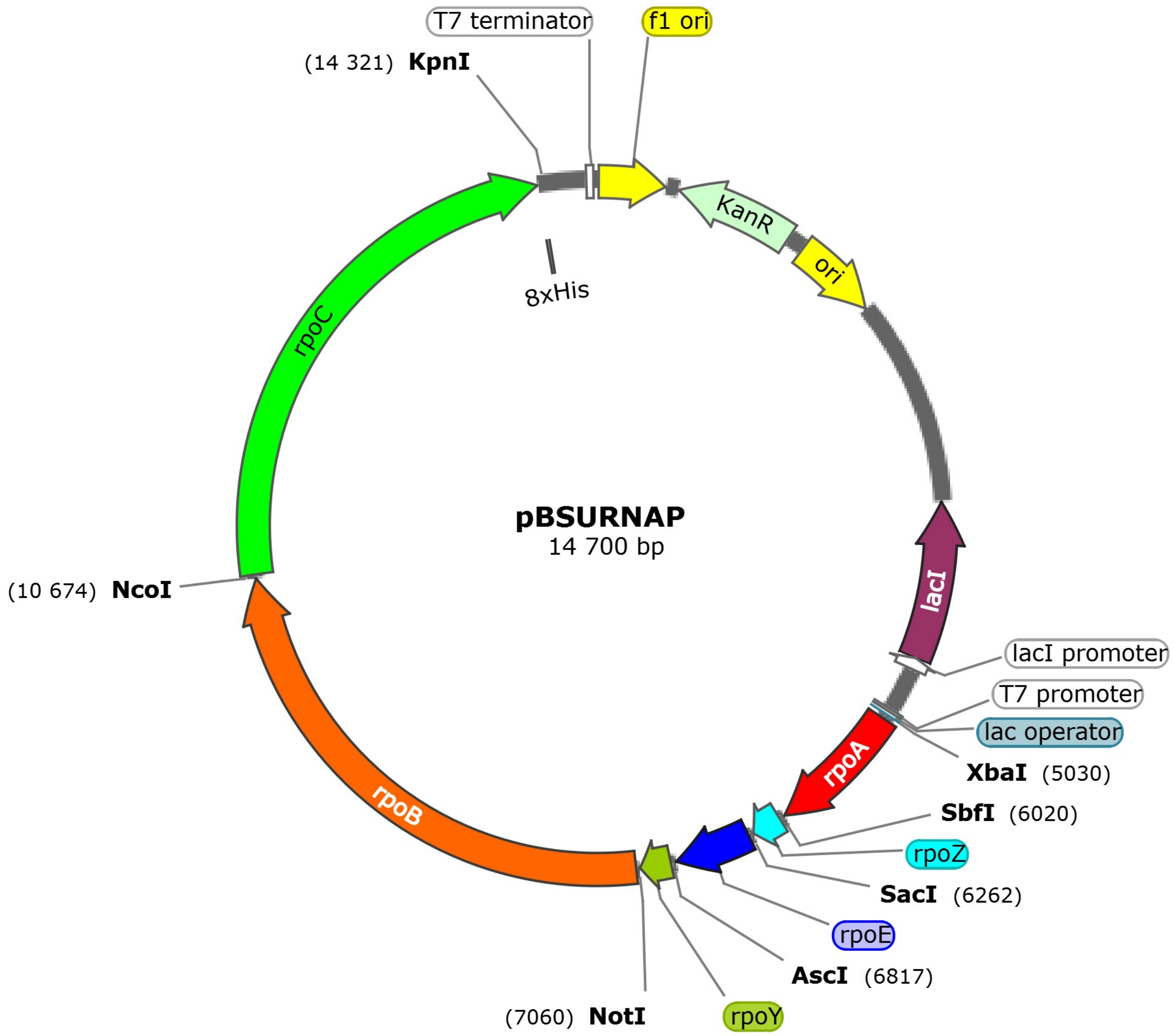

Figure S1. Plasmid map of pBSURNAP used for the expression of the core RNA polymerase.

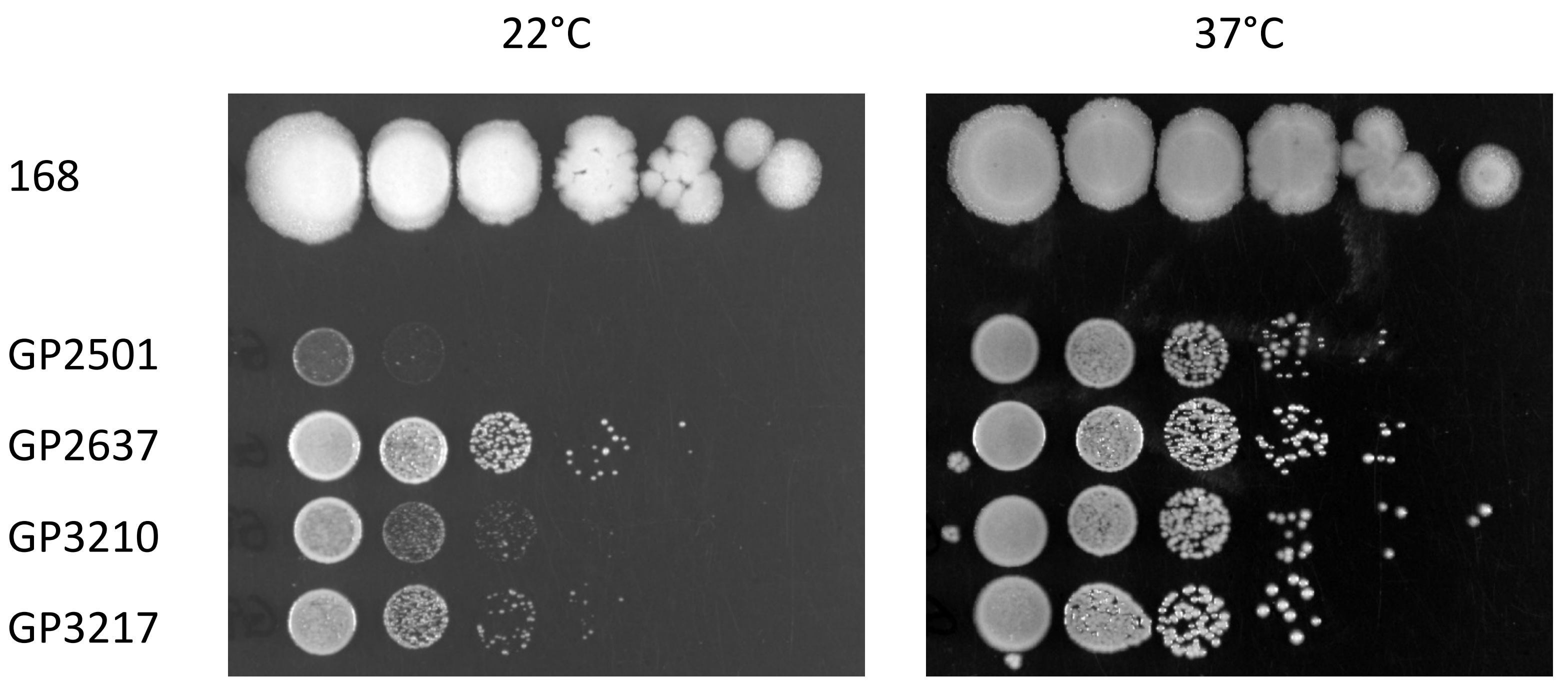

Genotypes:

GP2501: Δ*rny*

GP2637: Δ*rny* adeR$^{(Tyr55Asn)}$ *rpoE*$^{\Delta 199\text{-}208}$ Δ*skin* (*rrnW-rrnI*)$_2$

GP3210: Δ*rny rpoE* $^{(Insertion\ A88)}$

GP3217: Δ*rny* Δ*rpoE*

Figure S2. Suppressors of *rny* with mutations in *rpoE* and the *rny rpoE* double mutant show improved growth at 22°C, but not at 37°C.

Serial drop dilutions comparing growth of the wild type 168, the *rny* mutant (GP2501), its derived suppressor mutants evolved at LB agar plates at 22°C (GP2637 and GP3210) and the *rny rpoE* double mutant (GP3217). The pictures were taken after 3 days of incubation at 22°C and 1 day of incubation at 37°C, respectively.

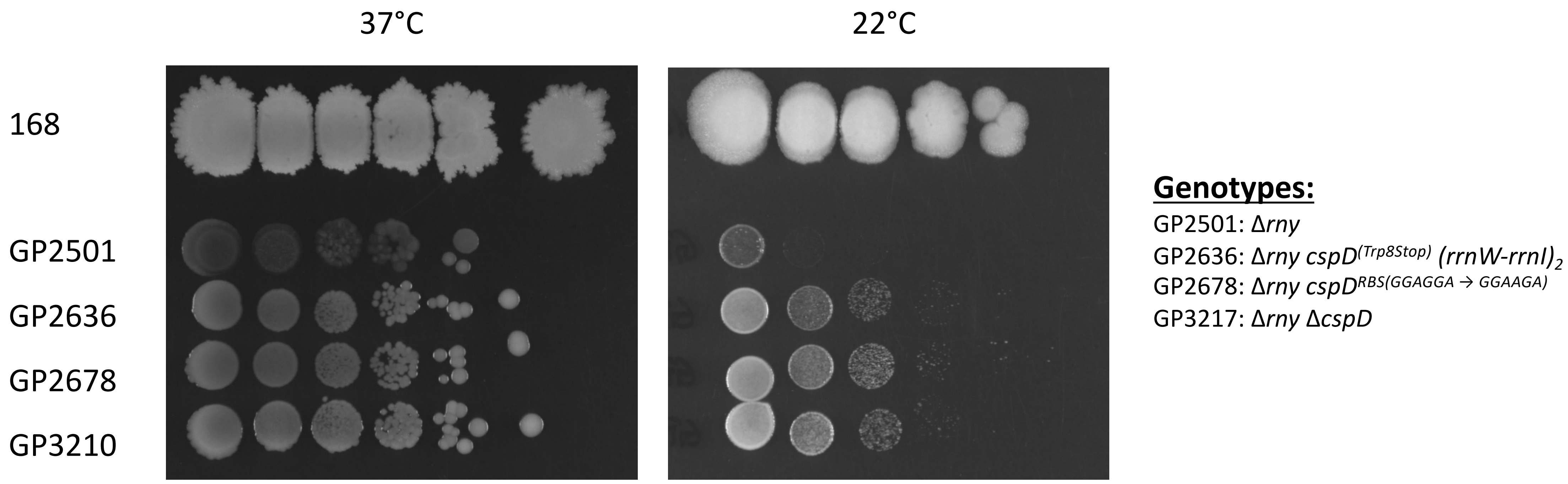

Figure S3. Suppressors of *rny* with mutations affecting *cspD* and the *rny cspD* double mutant show improved growth both at 37°C **and 22**°C**.** Serial drop dilutions comparing growth of the wild type 168, the *rny* mutant (GP2501), its derived suppressor mutants evolved at LB agar plates at 37°C (GP2636 and GP2678) and the *rny cspD* double mutant (GP2615). The pictures were taken after 3 days of incubation at 37°C and 22°C, respectively.

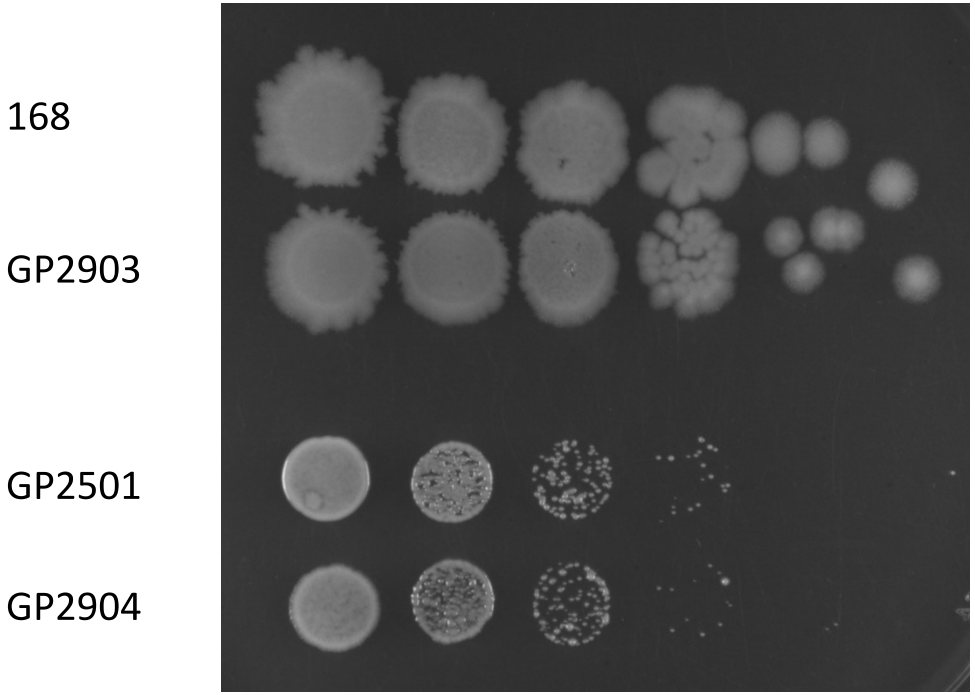

Figure S4. Relocation of *rpoA* does not affect growth. Serial drop dilutions comparing growth of the wild type 168, the wild type strain with relocated *rpoA* GP2903, and their respective *rny* deletion strains GP2501 and GP2904 on a LB plate at 37° C. The picture was taken after 18h of incubation.

Table S1. Oligonucleotides used in this study

**Primer**	**Sequence**<br>Restriction sites are underlined<br>His-tag sequences are italic<br>Homologous bases for joining PCR are bold
**Detection of *ctsR-pdaB* region duplication**	
MB30	5′-CTGTATGTCTTTGACCCCTAACTTTTC
MB208	5′-CCTCTTTCGCTTGTAAATCTGGT
**Control of *rny* deletion**	
NC9	5′-CTATGAAAAGATGTTTACGCCAGGG
ML101	5′-CTGCAAATTAATGACTGCTAGTTCTT
**Construction of pBSURNAP and its derivatives**	
LK#2684	5′-GGTCTAGAGCGGCCGCTTTAAGAAGGAGATATATCTATGACAGGTCAACTAGTTC
LK#2685	5′-CGCGGATCCGGTACCCCATGGCGCGCAAGTTCTTTTGTTACTACATCG
LK#2686	5′-GCGCCATGGTGGCTCGGGTGCAATGCTAGATGTGAACAATTTTGAG
LK#2687	5′-GCGGTACC*TTAGTGATGGTGATGGTGATGGTGATG*TTCAACCGGGACCATATCG
MB169	5′-AAAGCGGCCGCTTTAAGAAGGAGATATATCTATGACAGGTCAACTAGTTCAGTATGGAC
MB170	5′-AAACCATGGCGCGCAAGTTCTTTTGTTACTACATCGCGTTCAA
MB167	5′-AAACCATGGTGGCTCGGGTGCAATGCTAGATGTGAACAATTTTGAGTATATGAAC
MB168	5′-AAAGGTACC*CTAGTGATGGTGATGGTGATGGTGATG*TTCAACCGGGACCATATCGT
**Deletion of *cspD* gene**	
MB17	5′-CGCCGAACTGGAAGAGTCATTCC
MB18	5′-**CCTATCACCTCAAATGGTTCGCTG**GTTGAACCATTTTACTTTACCGTTTTGCAT
MB19	5′-**CCGAGCGCCTACGAGGAATTTGTATCG**GTAATCGTGGACCTCAAGCTTCTAATGTTG
MB20	5′-GAAGCACTCCTTGAATCGCTGAAGC
kan-fwd	5′-CAGCGAACCATTTGAGGTGATAGG
kan-rev	5′-CGATACAAATTCCTCGTAGGCGCTCGG

MB21	5′-GGCGAACTTGTCGATGAACATCAG
MB22	5′-GGCAGCTGGCCTTGTTATGATC
**Deletion of *rny* gene**	
ML47	5′-GAAGAATCTGCTTACACATACATCG
KG409	5′-**GACTGTGTTTTATATTTTTCTCGTTCAT**ACTTTCACCTCCTCTTGCTATGAACT
KG410	5′-**CCGAGCGCCTACGAGGAATTTGTATCG**AGTGATGCGCTAAGCATCACTTTATTTTTTTG
NP60	5′-GCAGACACATACTCTCCCACTTTTACACTGCTGACAT
KG411	5′-**ATGAACGAGAAAAATATAAAACACAGTC**
CZ68	5′-**CGATACAAATTCCTCGTAGGCGCTCGG**TTACTTATTAAATAATTTATAGCTATTG
KG414	5′-GTCGGTTCATCACAAAAAGCGCTGAT
NP61	5′-AGTATTGGTACACACATGAGATTTTCCTGTTAG
NC16	5′-CTGCCACTGAATTTGGACTCG
ML101	5′-CTGCAAATTAATGACTGCTAGTTCTT
**Construction of the strain GP2909 with relocated *rpoA* gene**	
SW17	5′-GACATTGTCCCTTTATCAGC
SW18	5′-**GGGGTGTGAGCTGAATTC**CTGCTGTCTGATCAATTTAATG
SW19	5′-**CCGAGCGCCTACGAGGAATTTGTATCG**CCCCCATGAAAAAAAGAC
SW20	5′-CGAATCAAATGCTTATTTGG
SW21	5′-GAATTCAGCTCACACCCC
SW40	5′-**TGGTTTTTCAATCTCGAT**CATTATTTTCCCTCCTTTTC
SW23	5′-ATGATCGAGATTGAAAAACCA
SW24	5′-**CCTATCACCTCAAATGGTTCGCTG**TCAATCGTCTTTGCGAAG
cat-fwd (kan)	5′-**CAGCGAACCATTTGAGGTGATAGG**CGGCAATAGTTACCCTTATTATCAAG
cat-rev (kan)	5′-**CGATACAAATTCCTCGTAGGCGCTCGG**CCAGCGTGGACCGGCGAGGCTAGTTACCC
SW25	5′-GATCATAATCTTCAATGCGAAG
SW27	5′-GAACAACCACAAATGACATC

SW28	5′-GTGATCTGTGAAAATCCAAAG
SW29	5′-**CCTATCACCTCAAATGGTTCGCTG**TACTTAAAACCCTCCTTCAAAAC
SW30	5′-**ATATTTTACTGGATGAATTGTTTTAGTAA**CTAGTTTCCCTTGTGAACTAGG
SW31	5′-CAACTCTCTGCTTTTGGC
kan-fwd	5′-CAGCGAACCATTTGAGGTGATAGG
kan-rev w/o T.	5′-TTACTAAAACAATTCATCCAGTAAAATAT
SW32	5′-GCGATGTTCAAAGTTGAAC
SW33	5′-CATATTTTTTACCGCCATTCA
SW41	5′-TTGTCAAGTGAAGGCGCGCTAT
mls-rev (kan)	5′-**CGATACAAATTCCTCGTAGGCGCTCGG**GCCGACTGCGCAAAAGACATAATCG
SW42	5′-AGTGAAGGAAAAGGGATG
SW43	5′-**CCGAGCGCCTACGAGGAATTTGTATCG**GTTCTGTAGATTCACTCCGA
SW44	5′-**ATAGCGCGCCTTCACTTGACAA**GAGACTTAGATTAAGTTGACGC
SW45	5′-ACTGTCAATATAGCATAAATTCC
**Construction of the strain GP3289 with duplicated *rpoA* and *rpoBC* genes**	
MB203	5′-GTTTGGTTCATACACTTGCGTTC
MB209	5′- CCGTCGTTCAAAAAAAGAAAGGC
MB210	5′-**CCTATCACCTCAAATGGTTCGCTG**TTATTCAACCGGGACCATATCGT
MB226	5′-**CCTATCACCTCAAATGGTTCGCTG**TTCTCTCACGAACCACACGC
MB240	5′-GATTTCACGTTATCCCGAATGCC
MB241	5′-**GCCTTTCTTTTTTTGAACGACGG**CTATTTTAAAATTAGCTGGTCCAACGC
MB242	5′-**CCGAGCGCCTACGAGGAATTTGTATCG**GTTTGAACAGGTCTTGTCATGGG
MB243	5′- GACCTATACAACCGATACGTACG
**Construction of plasmid pGP2542**	
KG412	5′-AAAGGATCCATGGCACAAGAGAAAGTTTTTCCTATG
KG413	5′-TTTGTCGACTGAAATTTTCACAATTTTCACGAGCATTTC

**Construction of plasmid pLK502**	
LK#125	5′-GGGAATTCATGGATTGCAAGATGATCTG
LK#127	5′-CCAAGCTTAGACCGAACTCATATTACGCCGC
**Construction of pGP2825 for CRISPR editing** [1]	
SW69	5′-AAGGCCAACGAGGCCCTTACTCACTTGTTAC
SW70	5′-AAGGCCTTATTGGCCTCTTGAAGCATACG
SW73	5′-aAGgATGGGaCACATTGAACTGGCTG
SW74	5′-tCCCATcCTtTCACGAtGGACTTTAGC
SW93	5′-(p)TACGTAAAGTCCGTCGTGAGAGAA
SW94	5′-(p)AAACTTCTCTCACGACGGACTTTA
**Construction of pGP2826 for CRISPR editing** [1]	
SW81	5′-AAGGCCAACGAGGCCCAAGCACAGCAAGTGATT
SW82	5′-aATGTTGTAgCCaTCgACcAACAGGATATCCATGGGT
SW83	5′-gGTcGAtGGcTACAACATtGATTGGAGCC
SW84	5′-AAGGCCTTATTGGCCCCTGAACAATATCCTCTCTG
SW85	5′-(p)TACGTGGATATCCTGTTAGTAGAC
SW86	5′-(p)AAACGTCTACTAACAGGATATCCA
**Sequencing primers**	
cspD	
KG227	5´- GAAGGAATCAGAAATGATGACCGCCA
KG228	5´- CGCTGTTTCCACCGCTAGTTCCA
rpoE	
MB5	5´-CACGCAAATCTATGAAGGCACTC
MB6	5´-GCTACAATACCCTTTCCAAGTGAG
adeR	
MB206	5´-GTCTTGCCTCCGATGACTTTC

MB207	5´-GCGCCTGTTTCAACCAGCA
greA	
KG384	5´-GAACGAGGACTGCCCTGTGTTCTC
KG385	5´-CTGCCAGCTTCATTCGTTTCGATATCTTC
rpoB	
MB9	5´-GAAGGCGTATCTGAGCGTGACG
MB176	5´-GAATCGCCTCTTCAATCAGAGAC
MB177	5´-TGGATGTATCGCCTAAGCAGGTT
SW63	5´-GATTCTTCCTGAAGAGGATATG
KG422	5´-GGATCAGTTACAACGTAAGAAGC
rpoC	
MB108	5´-GCGCTCAATTGTTTCAGTTCCTTC
MB175	5´-CAATTGTCCCGCAGTATAAGCTG
SW77	5´-GATACCGCTCTTAAAACTGC
SW87	5´-GAAACAAGCCTTCTTGGA
SW88	5´-CGTACCATCACGTATGAAC
purT-mpr region	
MB245	5´-GGACCCAGAGCAGATCAGAA
MB246	5´-GAACAAGGGTGACGAACGATG
**pJOE8999**	
SW79	5′-GAATACCGGTTGCATCTG
SW80	5′-AGATTATTGAGCAAATCAGTG
**pGP2542**	
M13fwd	5′-GTAAAACGACGGCCAGTG

[1] Phosphorylated primers are indicated with (p). Mutation position are written in lower case.

Table S2. Effect of the *rny* deletion on the expression of *B. subtilis* genes and operons.

All operons that exhibited an at least eight-fold change upon deletion of *rny* are shown (and relevant sigma factor genes). In case of differential expression within one operon, the genes not in bold did not met this 8-fold criteria.

Transcription unit	Function [a]	Regulation [b]	Fold changes
mRNAs with increased amount in the *rny* mutant			
yxkC	unknown	SigD, TnrA	**0.010**
epr	minor extracellular serine protease, involved in control of swarming motility	SigD, Spo0A, SinR, DegU, ScoC	**0.013**
yfmT-S	vanillin dehydrogenase/soluble chemotaxis receptor	SigD	**0.022**
motA-B	H+-coupled MotA-MotB flagellar stator	SigD	**0.023**
hemAT	soluble chemotaxis receptor, heme-containing O_2 sensor protein	SigD	**0.026**
lytF	major autolysin	SigD, SinR, SlrR	**0.031**
hag	flagellin protein	SigD, CodY, ScoC, CsrA	**0.031**
glpT-Q	glycerol-3-phosphate permease and diesterase	GlpP, PhoP, CcpA	**0.036**
pyrR-***P-B-C-AA-AB-K-D-F-E***	pyrimidine biosynthesis	PyrR	**0.047**
pgdS	gamma-DL-glutamyl hydrolase	SigD	**0.049**
pstS-C-A-BA-BB	high-affinity phosphate uptake	PhoP	**0.056**
artP-*Q-R*	high affinity arginine ABC transporter	YlxR	**0.056**
yvbX	putative glycoside hydrolase		**0.065**
yvbJ	unknown		**0.065**
lip	extracellular lipase	AbrB	**0.067**
yxeK-snaB-yxeM-N-O-sndB-yxeQ	N-acetylcysteine deacetylase	CymR	**0.068**
tlpA-***mcpA***	membrane-bound chemotaxis receptor, methyl-accepting chemotaxis protein	SigD, AbrB	**0.079**

yocC*-D*	unknown		**0.083**
ctaO	heme O synthase (minor enzyme)	AbrB	**0.083**
lytA-B*-C*	autolysins	SigD, SinR, YvrHb, SlrR	**0.090**
tlpC	membrane-bound chemotaxis receptor, methyl-accepting chemotaxis protein	SigD	**0.090**
mntA*-B-C-D*	manganese ABC transporter	MntR	**0.093**
tuaA/2-A/1-B-C-D-E-F-G-H	biosynthesis of teichuronic acid	PhoP, SigF	**0.096**
flhO-P	flagellar assembly	SigD	**0.102**
yvyC-fliD-S-T-A*-hpf*	flagellar assembly	SigD	**0.109**
*sunA-**sunT**-bdbA-yolJ-bdbB*	sublancin export and processing	Rok, AbrB, Abh, YvrHb, DnaA	**0.114**
natA*-B*	Na^+ ABC transporter (export)	NatR	**0.114**
spo0M	sporulation control	SigH, SigW	**0.122**
dgcW	synthesis of c-di-GMP	SigD	**0.122**
lipB	extracellular lipase		**0.123**
sigD	alternative sigma factor	SigD, Spo0A, SwrA, CodY, DegU	**0.430**
mRNAs with decreased amount in the *rny* mutant			
*yonP -O-**N***	SPβ prophage		**18.51**
sspF	small acid-soluble spore protein	SigG	**18.00**
yhdX	unknown		**16.37**
ysnF	general stress protein, survival of ethanol stress	SigB	**15.22**
sspB	small acid-soluble spore protein	SigG, SpoVT	**14.93**
yukJ	unknown		**14.52**
nhaX	general stress protein, putative regulator of *nhaC*	SigB	**14.03**

levD-E-F-G-*sacC*	fructose-specific phosphotransferase system	SigL, LevR, CcpA	**13.30**
yhfH	unknown	YlxR	**13.17**
yjbC-*spx*	general stress proteins, required for survival of salt and paraquate stresses	SigB, SigM, SigW, SigX, PerR	**12.81**
ytzE	transcriptional regulator		**12.21**
fbpB	RNA chaperone for *fsrA*, response to iron limitation	Fur	**12.03**
yrzF	putative serine/threonine-protein kinase		**11.73**
frlB-O-N-M-*frlD-yurJ*	Uptake and metabolisms of sugar amines	FrlR, CodY, YlxR	**11.00**
corA	general stress protein, similar to magnesium transporter	SigB , LexA	**9.84**
yocH	peptidoglycan hydrolase (amidase)	Spo0A, WalR, AbrB	**9.76**
speD	S-adenosylmethionine decarboxylase,	CcpN	**9.72**
mreBH-ykpC	cell shape-determining protein/unknown	SigI, WalR	**9.48**
slp	small peptidoglycan-associated lipoprotein		**9.36**
yrhH	similar to methyltransferase	SigW,SigM,SigX	**9.05**
tlrB	23S rRNA (guanine-N(1)-)-methyltransferase		**9.03**
yuzA	general stress protein	SigB, SigG	**8.61**
rsfA	Regulator of SigF-dep. transcription	SigF, SigG	**8.57**
bsrA-***yrvM***	6S RNA/ tRNA modification enzyme		**8.55**
yqhB	similar to magnesium exporter, general stress protein	SigB, LexA	**8.42**
sigF	sporulation-specific sigma factor	SigH, SigF, SigG, AbrB, SinR, Spo0A	**4.23**
sigG	sporulation-specific sigma factor	SigF, SigG, AbrB, SinR, Spo0A	**4.68**

[a] Information was taken from *Subti*Wiki database (Zhu and Stülke, 2018)

[b] The housekeeping sigma factor σ^A is not listed as a regulator

Table S3. Genes with (partially) restored expression in the suppressor mutant

Numbers of reads corresponding to the listed genes are shown.

#	Gene	Wild type 168	GP2501 Δ*rny*	GP2518 Δ*rny* Δ*greA* (*rrnW-rrnI*) $_2$
	**Genes upregulated in the *rny* mutant**			
1	*yonN*	14	265	107
2	*sspF*	33	594	245
3	*levE*	11	174	23
4	*sspB*	13	199	20
5	*yukJ*	211	3068	1378
6	*frlB*	49	714	100
7	*frlO*	17	221	25
8	*levF*	17	191	23
9	*levG*	20	173	36
10	*yuzA*	12	103	48
11	*rsfA*	12	106	19
12	*xtmB*	752	5972	259
13	*xkdE*	733	5440	222
14	*xtmA*	433	3171	128
15	*rocA*	1077	7435	529
16	*yonH*	13	86	28
17	*trpC*	31	197	89
18	*qdoI*	67	403	127
19	*yonJ*	24	143	34
20	*yfhK*	63	371	150
21	*yfiU*	50	277	83
22	*trpB*	80	433	215
23	*opuCA*	234	1219	222
24	*veg*	1810	9193	3024
25	*yxaH*	86	434	191
26	*xkdU*	143	687	38
27	*ydaD*	58	280	129
28	*ykgA*	32	151	64
29	*yrkF*	12	58	19
30	*pksD*	25	112	30
31	*yomV*	23	103	47
32	*opuCB*	109	474	80
33	*xkzA*	72	313	14
34	*oxdC*	94	401	130
35	*yjgD*	28	117	58
36	*ybbA*	278	1161	334
37	*yrkH*	28	118	47
38	*xkdR*	82	341	16
39	*ypzA*	30	125	46

40	*gerW*	25	101	27
41	*xkdM*	558	2273	120
42	*xkdS*	93	377	14
43	*xkdQ*	305	1222	61
44	*ykzL*	363	1442	62
45	*feuA*	656	2594	628
46	*xkdF*	749	2930	117
47	*xepA*	290	1121	39
48	*spoIIAA*	13	51	21
49	*xkdW*	114	437	17
50	*bacB*	51	196	88
51	*youA*	31	117	43
52	*xkdG*	914	3477	139
53	*yomW*	18	69	19
54	*opuCC*	165	618	103
55	*opuCD*	169	628	98
56	*yonB*	48	180	51
57	*xkdV*	534	1977	77
58	*ykzI*	15	55	25
59	*speA*	4381	16034	6933
60	*feuB*	446	1623	484
61	*murAA*	39336	142509	56216
62	*xkdH*	350	1269	59
63	*xkdP*	247	888	64
64	*rocD*	539	1933	455
65	*spoIIAB*	25	88	44
66	*yomU*	40	141	46
67	*yisT*	40	140	65
68	*xkdK*	1439	5052	229
69	*xkdT*	263	921	45
70	*yomX*	25	86	27
71	*xhlB*	155	539	19
72	*yonA*	30	104	31
73	*feuC*	419	1437	488
74	*xlyA*	795	2716	137
75	*xkdI*	461	1562	55
76	*yerD*	52	174	79
77	*bacC*	69	233	106
78	*xkdO*	1611	5392	722
79	*xkdJ*	364	1202	59
80	*pksE*	43	140	29
81	*yobO*	62	201	64
82	*yonD*	65	203	72
83	*xkdN*	295	863	51
84	*yonF*	15	45	17
85	*azoR2*	407	1181	518
86	*yddJ*	41	115	41
87	*yonC*	26	73	21
88	*yorG*	37	104	30
89	*gmuA*	277	766	53

90	*yorF*	30	83	14
91	*yisK*	335	922	408
92	*rocB*	119	323	56
93	*ykzM*	239	646	35
94	*yonO*	16	43	12
95	*spoIIT*	4930	13052	5899
96	*gmuD*	1313	3471	215
97	*gltB*	936	2468	987
98	*xhlA*	248	651	21
99	*yomE*	18	46	12
100	*yybF*	464	1212	436
101	*yonE*	35	87	41
102	*tagB*	1918	4643	1720
103	*yosP*	51	121	38
104	*tagA*	2932	6995	2221
105	*nrdEB*	40	94	38
106	*yqgY*	282	659	325
107	*spoVG*	2028	4548	1825
108	*cwlS*	222	495	118
109	*bacD*	193	429	195
110	*yomM*	16	36	16
111	*bdhA*	5374	11288	2499
112	*opuD*	2318	4837	2384
113	*galM*	80	165	79
114	*gmuR*	749	1533	212
115	*yorI*	24	48	22
	**Genes downregulated in the *rny* mutant**			
1	*artP*	489	27	57
2	*sndB*	836	68	265
3	*yvyC*	447	39	94
4	*fliD*	4264	374	777
5	*yxeQ*	935	103	319
6	*spo0M*	7850	954	2678
7	*epsD*	185	25	52
8	*cydA*	83	13	81
9	*nrgA*	404	63	359
10	*xpt*	378	68	141
11	*epsN*	61	12	28
12	*ywlD*	232	45	132
13	*yxeR*	1218	237	478
14	*yteJ*	2911	579	1208
15	*qdoR*	1982	436	1148
16	*skfB*	74	17	35
17	*yxjI*	781	190	424
18	*yvaV*	1228	311	757
19	*tsaC*	1244	320	683
20	*yjoB*	2355	606	1312
21	*pucR*	235	64	173
22	*nasB*	43	13	41

23	*cypA*	101	30	107
24	*exoA*	454	138	402
25	*ybaE*	2328	739	1821
26	*ydgG*	94	30	73
27	*hmp*	92	29	86
28	*yqaS*	45	15	163
29	*hisZ*	38	13	47
30	*yqaT*	46	16	131
31	*yqbB*	45	15	72
32	*yoyA*	74	25	61
33	*hisD*	63	22	58
34	*comFA*	142	52	146
35	*hisA*	82	31	97
36	*hutU*	98	37	95
37	*spoIIB*	42	16	42
38	*fra*	559	216	551
39	*cydB*	81	31	77
40	*hisF*	102	40	100
41	*phoD*	75	31	65
42	*proI*	584	244	1099
43	*ywpB*	2387	999	2194
44	*hutI*	111	47	103
45	*hisH*	39	16	53
46	*fosB*	352	150	302
47	*alaT*	4956	2120	5504
48	*yclG*	142	61	127
49	*leuB*	61	26	56
50	*yqaR*	73	32	128
51	*bofA*	235	102	252
52	*cydC*	220	96	211
53	*spsB*	26	11	27
54	*alaR*	1460	647	1733
55	*yrpG*	46	20	68
56	*fnr*	391	175	562
57	*trmFO*	3176	1438	3014
58	*gapB*	268	130	294
59	*hisB*	37	18	39
60	*yqbA*	45	22	100
61	*spsG*	39	19	41
